# Detection of Aortic Calcification during Vertebral Fracture Assessment (VFA) Compared to Digital Radiography

**DOI:** 10.1371/journal.pone.0000715

**Published:** 2007-08-08

**Authors:** John T. Schousboe, Kevin E. Wilson, Thomas N. Hangartner

**Affiliations:** 1 Park Nicollet Health Services, Minneapolis, Minnesota, United States of America; 2 Division of Health Policy and Management, School of Public Health, University of Minnesota, Minneapolis, Minnesota, United States of America; 3 Hologic, Bedford, Massachusetts, United States of America; 4 BioMedical Imaging Laboratory, Wright State University and Miami Valley Hospital, Dayton, Ohio, United States of America; Community Information and Epidemiological Technologies, Canada

## Abstract

**Background:**

Cardiovascular disease is the most common cause of mortality among post-menopausal women. Our objective was to determine whether or not lateral spine images obtained on a bone densitometer to detect prevalent vertebral fracture can also accurately detect radiographic abdominal aortic calcification (AAC), an important risk factor for cardiovascular disease independent of clinical risk factors.

**Methodology/Principal Findings:**

One hundred seventy four postmenopausal women had bone densitometry, lateral spine densitometry imaging (called vertebral fracture assessment, or VFA), and lateral spine digital radiographs. Radiographs and VFA images were scored for AAC using a previously validated 24 point scale and a simplified, new 8 point scale (AAC-8). One hundred fifty six (90%) of the VFA images were evaluable for AAC. The non-parametric intraclass correlation coefficient between VFA and radiographic 24 point and AAC-8 readings, respectively, were 0.80 (95% C.I. 0.68–0.87) and 0.76 (95% C.I. 0.65–0.84). Areas under receiver operating characteristics (ROC) curves for VFA to detect those with a radiographic 24-point AAC score ≥5 were 0.86 (95% C.I. 0.77–0.94) using the 24 point scale and 0.84 (95% C.I. 0.76–0.92) using the AAC-8 scale.

**Conclusion/Significance:**

VFA imaging intended to detect prevalent vertebral fracture can also detect radiographic AAC, an important cardiovascular disease risk factor. Since bone densitometry is recommended for all women age 65 and older, VFA imaging at the time of bone densitometry offers an opportunity to assess this risk factor in the post-menopausal female population at very little incremental time and expense.

## Introduction

Coronary heart disease and stroke, respectively, are the first and third leading single causes of mortality among elderly women.[1] Over 60% of women who die of coronary heart disease have no prior symptoms of the disease.[2] Identification of those at risk of cardiovascular disease and for whom aggressive preventive measures should be directed has relied on clinical risk factors such as hypertension, cigarette smoking, obesity, family history, and diabetes mellitus.[3] However, 40% of the population is at intermediate risk when judged by these risk factors, and it is unclear just how aggressively their modifiable risk factors, such as LDL cholesterol, should be treated.[4] A variety of imaging studies to detect subclinical cardiovascular disease, including lateral spine radiography, have been proposed to improve identification of those who would benefit from more aggressive treatment of risk factors such as LDL cholesterol and blood pressure. Abdominal aortic calcification scored semi-quantitatively with a 24 point scale on lateral lumbar spine radiographs is predictive of cardiovascular disease incidence and mortality independent of other clinical risk factors.[5]–[8]

Osteoporosis is also a very common condition among elderly women, and universal bone densitometry is now widely recommended for all women age 65 and older.[9]–[12] Simultaneous lateral spine imaging is also recommended for a sizable subset of the elderly female population to detect prevalent vertebral fracture, a powerful predictor of subsequent fractures independent of bone density.[13], [14] Since the average age of first myocardial infarction in women is 70.4 years,[15] detecting sub-clinical atherosclerosis in the population eligible for bone densitometry has the potential to allow for better stratification of risk for those at intermediate risk of cardiovascular disease.

It has recently been shown in a small pilot study that lateral spine images obtained with X-ray densitometry to detect prevalent vertebral fracture (VFA) can detect abdominal aortic calcification (AAC) with reasonably good sensitivity and specificity.[16] However, in that pilot study only 28% of VFA's fully contained the region of the abdominal aorta because the technicians acquiring the VFA's were not instructed to include that region. Also, the radiographs were read on non-digital films rather than digital electronic radiographic images, and the population was not specifically selected to be at high risk for prevalent vertebral fracture.[16]

The aims of the present investigation were to test the hypothesis that a high percentage of VFA's would include the abdominal aorta if the technician was specifically instructed to include this region during VFA acquisition, and to re-examine how well VFA images detect AAC relative to lateral abdominal digitized electronic radiographic images in a larger, new cohort of post-menopausal women at high risk for vertebral fracture.

## Methods

This study was funded by Hologic, Inc. (Bedford, MA), and was approved by the institutional review board of Miami Valley Hospital in Dayton, Ohio. Informed signed consent was obtained for all study participants.

### Study population

Study subjects eligible to participate were post-menopausal women age 55 and older who were able to give informed consent for participation. To select a population at high risk for vertebral fracture for whom a VFA exam would be indicated in clinical practice, eligible participants had to have a prevalent vertebral fracture index (PVFI) of ≥3, or a PVFI = 2 combined with a femoral neck T-score of −2.5 or lower. The PVFI is an index of prevalent vertebral fracture probability, based on age, self-reported fracture history, diagnosis of osteoporosis, and self-reported height loss.[17] Those who were non-ambulatory, with a body mass index >35 kg/m^2^, or with self-reported scoliosis were excluded.

Five hundred twenty women residing in Dayton, Ohio, responded to study advertisements, and 509 of these women were contacted by phone and expressed interest in participation. Initial screening by phone excluded 307, who did not meet study inclusion and exclusion criteria. One hundred ninety five came for an initial study visit and had baseline demographic data collected and bone densitometry done, but 19 were judged at that point not to meet the study inclusion criteria. Of the remaining 176, two did not have lateral lumbar spine radiographs done, leaving 174 study participants for this report.

### Study Procedures

Lateral single-energy (VFA) images of the thoraco-lumbar spine were obtained on a Hologic Discovery densitometer (Hologic, Inc., Bedford, MA) in the lateral decubitus position.

Lateral digital radiographs of the lumbar spine were obtained on that same day with the beam focused on L3, at a tube to film distance of 100 cm. The VFA images were scored twice for AAC by one person (JTS) using the Hologic Physician Viewer software, first using a 24-point AAC scale (detailed below) and a few days later using a simplified 8-point scale (AAC-8). One month later, the digital lateral spine radiographs were assessed twice by that same person for AAC using Image J software from the National Institutes of Health,[18] first using the 24 point scale and a few days later using the 8 point scale while blinded to his previous 24 point scale reading.

To ensure that the reader was blinded to his AAC readings on the opposite technology, the VFA images and radiographs were given separate study numbers for each person, and the code linking those readings to each study participant was withheld from the reader. Finally, one month after the radiographic readings were completed, the VFA images were re-read using the AAC-8 scale in order to estimate its intra-observer reliability. The inter-rater reliability has already been established for the 24-point scale on VFA in a previous study.[16]

### Scoring of AAC

Details of the 24 point AAC scale have been published elsewhere.[19] Briefly, the anterior and posterior aortic walls were divided into four segments, corresponding to the areas in front of the lumbar vertebrae L1–L4. Within each of these 8 segments, aortic calcification was recognized visually as either a diffuse white stippling of the aorta extending out to the anterior and/or posterior aortic walls, or as white linear calcification of the anterior and/or posterior walls. Aortic calcification scored as 0 if there was no calcification, as 1 if one-third or less of the aortic wall in that segment was calcified, as 2 if more than one-third but two-thirds or less of the aortic wall was calcified, or as 3 if more than two-thirds of the aortic wall was calcified. The scores were obtained separately for the anterior and posterior aortic wall, resulting in a range from 0 to 6 for each vertebral level and 0 to 24 for the total score.

The AAC-8 scale estimates the total length of calcification of the anterior and posterior aortic walls in front of vertebrae L1 to L4 as 0 if no calcification is seen, as 1 if the aggregate length of calcification is equal to the height of one vertebrae or less, 2 if that length is more than 1 but less than or equal to the heights of two vertebrae, 3 if that length is more than two but less than or equal to the heights of three vertebrae, and 4 if the aggregate length of calcification is more than the height of three vertebrae. Both the anterior and posterior walls are scored from 0 to 4, and the total score range is 0 to 8.

### Statistical Analysis

All analyses were done using statistical software Stata SE9. Because the distributions of both 24 point and 8 point AAC scales are highly skewed, agreement between scores on digital radiographs and VFA images were estimated with the intraclass correlation coefficient using a non-parametric bootstrap method with 1,000 repetitions.[20] Since a 24-point AAC scale score of ≥5 has been shown previously to be associated with a 2.4 fold increased risk of cardiovascular disease mortality,[7], [8] receiver operating characteristics (ROC) curve analyses[21] were done to assess how well the AAC-8 radiographic scores, the 24-point VFA scores, and the AAC-8 VFA scores identify those with a radiographic AAC-24 point scale score ≥5. Diagnostic tests with ROC curve areas between 0.70 and 0.90 relative to a gold standard are considered to have acceptable predictive accuracy, and those with ROC curve areas >0.90 are considered to have superb predictive accuracy.[21] The intra-observer reliability of the AAC-8 scale VFA score was estimated as the intraclass correlation coefficient between the two AAC-8 scores on VFA, again using a non-parametric method.

## Results

**Figure 1:**
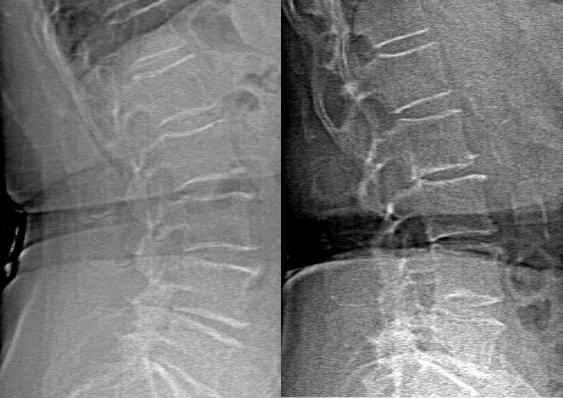
Examples of VFA Images with Inadequate Space (Left Panel) and Adequate Space (Right Panel) Anterior to the Aorta for AAC Assessment.

One hundred fifty six (90%) of the VFAs visualized sufficient space anterior to the lumbar spine to contain the entire abdominal aorta (**figure 1**). The mean age, body mass index, and ethnicity of participants with both radiographs and VFA images evaluable for AAC were equivalent to the 39 who did not have evaluable radiographs or VFA images or who were excluded at their first study visit (**table 1**). Those participants with fully evaluable images for AAC had a significantly higher total hip BMD T-score and trends for higher T-scores at the femoral neck and lumbar spine, compared to non-participants.

**Table 1:** Baseline characteristics of study population.

Variable	Evaluable AAC (n = 156)	Non-Evaluable AAC (n = 39)
Mean Age, years (Standard Deviation)	68.7 (7.9)	68.9 (8.2)
Mean Body Mass Index, kg/m^2 ^(SD)	27.0 (3.9)	27.5 (5.2)
Ethnicity		
White	94.8%	91.9%
Black	5.2%	8.1%
Mean Total Hip T-Score* (SD)	−0.97 (1.05)	−1.37 (0.99)
Mean Femoral Neck T-Score (SD)	−1.43 (0.83)	−1.64 (0.71)
Mean Lumbar Spine T-Score (SD)	−0.74 (1.45)	−1.15 (1.75)

* p-value for difference between groups 0.034

**Figure 2:**
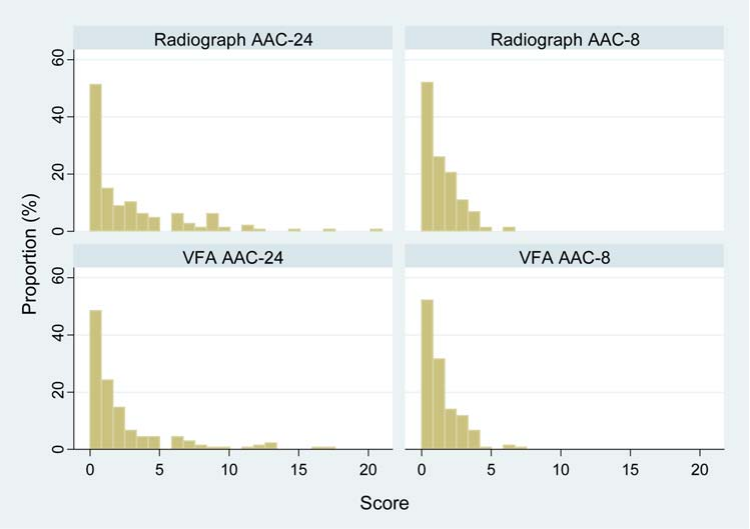
Frequency Distributions of 24-Point and AAC-8 Scores on Radiographs and VFA Images.

Histograms of the AAC scores on radiographs and VFA images showed that from 41% (with the 24-point scale on VFA) to 44% (with AAC-8 scale on either VFA or radiographs) of the evaluable participants did not have any detectable AAC (**figure 2**). Conversely, the prevalence of significant atherosclerotic burden, defined as a radiographic 24-point AAC score of 5 or higher, was 17%.

**Figure 3:**
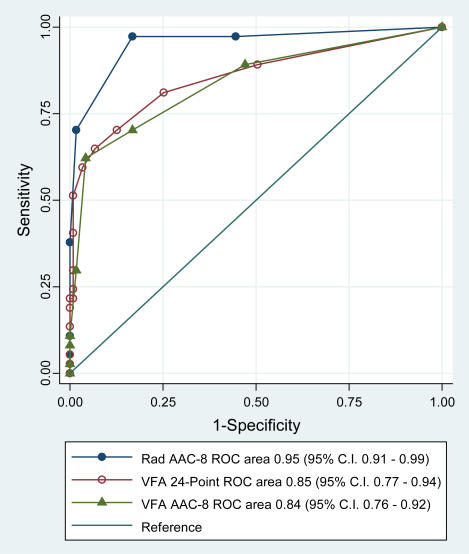
Receiver Operating Curve (ROC) Areas for Detection of Those with a Radiographic 24-point AAC score of 5 or Higher.

The intraclass correlation coefficient (ICC) between the 24 point AAC scores with radiography and VFA was 0.80 (95% C.I. 0.68–0.87), whereas the ICC between the AAC-8 radiographic and VFA scores was 0.76 (95% C.I. 0.65–0.84). Considering the 24 point radiographic readings as the “gold standard”, receiver operating characteristics curves showed, as expected, a very high area under the curve for the radiographic AAC-8 score, with modestly lower areas under the curve for the 24 point and AAC-8 readings on VFA images (**figure 3**). By this criterion, the AAC-8 scale performed as well as the 24 point scale on VFA images in distinguishing those with a radiographic 24 point scale AAC score of 5 or higher.

On VFA, AAC-8 and 24-point scale cutpoints, respectively, of ≥3 and ≥5 had the best sensitivity while maintaining specificities of 95% or higher. An AAC-24 score of 5 or higher on VFA had a sensitivity and specificity, respectively, of 59% and 97% of identifying those with a radiographic AAC-24 score of 5 or higher. The positive and negative predictive values of an AAC-24 score of 5 or higher on VFA for a 24 point score of 5 or higher on radiographs, respectively, was 85% and 88%. Similarly, an AAC-8 score of 3 or higher had a sensitivity and specificity, respectively, of 62% and 96% of identifying those with a radiographic 24 point AAC scale score of 5 or higher, with positive and negative predictive values of 82% and 89%. AAC scores of *zero* on VFA with either scale had a negative predictive value of 94% for a radiographic 24 point AAC score of 5 or higher.

Agreement with a radiographic 24 point AAC score of 5 or higher was moderate on VFA for both an AAC-8 score of 3 or higher (kappa 0.63, 95% C.I. 0.55–0.71) and an 24 point scale score of 5 or higher (kappa 0.62, 95% C.I. 0.55–0.70). The intra-class correlation between the initial and repeat AAC-8 scores on VFA was 0.87 (95% C.I. 0.80–0.92).

## Discussion

Radiographic AAC has been shown to be significantly predictive of overall cardiovascular disease incidence and mortality,[8] coronary heart disease,[7], [8] stroke,[5] congestive heart failure,[6] and intermittent claudication,[22] independent of clinical risk factors such as blood pressure, total and HDL cholesterol, smoking status, body mass index, and the presence of diabetes mellitus. The results of this study demonstrate that single-energy lateral spine imaging on a bone densitometer, intended primarily to detect individuals with prevalent vertebral fracture, can also reliably identify those with radiographic AAC, but with far less radiation exposure than standard radiographs (5 µSv for VFA compared to 600 µSv for lateral spine radiography).[23]

Cardiovascular disease remains the leading cause of mortality among elderly women, and the strategy of identifying those requiring more intensive therapy for treatable risk factors (such as blood pressure and hyperlipidemia) through the use of clinical risk factors alone is increasingly being viewed as suboptimal.[4], [24]–[26] Imaging studies such as electron beam computed tomography (EBCT) or CT coronary angiography are increasingly being used in addition to clinical risk factors to identify those who are at high risk of incident coronary heart disease.[27], [28] Radiographic AAC is also highly predictive of coronary artery calcification detected by EBCT,[27] and is significantly correlated with the presence of generalized atherosclerotic cardiovascular disease.[27], [29]

Bone densitometry is widely recommended for all women age 65 and older.[9]–[12] Simultaneous lateral spine imaging is now also recommended for a sizable subset of the elderly female population to detect prevalent vertebral fracture[13], [14] and has been shown to be cost-effective for that subset.[14] Sufficient soft tissue is highly likely to be visualized on the VFA image to allow evaluation for AAC, if the technician obtaining the image is instructed to include sufficient soft tissue anterior to the lumbar vertebrae. While AAC is not as predictive of coronary heart disease as EBCT,[30] the marginal expense and time requirement to obtain and interpret VFA images for AAC is minimal, particularly if AAC is scored using the AAC-8 scale which in our experience requires less than one minute. The intra-rater reliability of the AAC-8 scale, although slightly lower than what has been reported previously for the 24 point scale,[19] is still good.

Simultaneously identifying an important cardiovascular disease risk factor would improve the utility of this technology for this population even further. Osteoporosis is also associated with incident cardiovascular disease,[31] and hence delineation of cardiovascular disease risk may be particularly important for women referred for bone densitometry. We believe, therefore, that it is reasonable for those who provide bone densitometry services with vertebral fracture assessment to instruct their technicians to attempt to visualize adequate space anterior to the lumbar spine such that AAC can be assessed.

Regardless of whether or not assessments for AAC are planned, physicians who order and/or interpret VFA images to detect prevalent vertebral fracture should be aware of the association between AAC and incident cardiovascular disease, and not ignore its presence. In particular, it may be appropriate to review a patient's cardiovascular disease risk factor management and consider whether or not additional coronary artery imaging such as CT angiography is indicated if their VFA image shows a 24 point scale AAC score of ≥5 or an AAC-8 score of ≥3. More aggressive treatment of modifiable risk factors such as hypercholesterolemia may be indicated for these individuals.

Our study has several important strengths. It is the first study with a pre-planned aim of assessing the accuracy of AAC detection on VFA images compared to radiography, a well-established gold standard technique of AAC detection. This is also the first study to compare AAC detection on VFA images compared to digital lateral spine radiographs. Second, the reader of these images with either scale or technology was blinded to his readings using the opposite scale and to his readings on the opposite technology. Thirdly, the study was done in a population at high risk of vertebral fracture, such that VFA imaging is medically appropriate. Fourth, this is the first study to assess the intra-rater reliability of the AAC-8 scale.

There are also important limitations to this study. The prevalence of AAC was lower than expected in our study, which may reflect healthier individuals being more willing to participate in this study compared to the post-menopausal female population at large. Whereas this study implies an indirect association with prevalent cardiovascular disease, the direct link between AAC on VFA images and incident cardiovascular disease remains to be established. These results are applicable only to post-menopausal women. Finally, densitometric vertebral fracture assessment currently is covered only in the United States, although if further studies confirm the utility of this procedure in post-menopausal women, perhaps more widespread coverage of the procedure will follow.

In summary, VFA imaging with a bone densitometer can simultaneously detect prevalent vertebral fracture and abdominal aortic calcification an important cardiovascular disease risk factor. Since bone densitometry is indicated for all women age 65 and older, VFA imaging has the potential to contribute to identification of sub-clinical cardiovascular disease in the post-menopausal female population at large. At the least, clinicians should be aware of the associations between AAC on VFA images and radiographs, and between radiographic AAC and incident cardiovascular disease. If significant AAC is noted on a VFA image, follow-up assessment of the patient's overall cardiovascular disease risk management is indicated.

## References

[1] Kochanek KD, Smith BL (2002). Deaths: Preliminary Data for 2002.. National Vital Statistics Report.

[2] Wenger NK (2003). Coronary heart disease: the female heart is vulnerable.. Prog Cardiovasc Dis.

[3] Greenland P, Knoll MD, Stamler J, Neaton JD, Dyer AR (2003). Major risk factors as antecedents of fatal and nonfatal coronary heart disease events.. Jama.

[4] Greenland P, Smith SC, Grundy SM (2001). Improving coronary heart disease risk assessment in asymptomatic people: role of traditional risk factors and noninvasive cardiovascular tests.. Circulation.

[5] Hollander M, Hak AE, Koudstaal PJ, Bots ML, Grobbee DE (2003). Comparison between measures of atherosclerosis and risk of stroke: the Rotterdam Study.. Stroke.

[6] Walsh CR, Cupples LA, Levy D, Kiel DP, Hannan M (2002). Abdominal aortic calcific deposits are associated with increased risk for congestive heart failure: the Framingham Heart Study.. Am Heart J.

[7] van der Meer IM, Bots ML, Hofman A, del Sol AI, van der Kuip DA (2004). Predictive value of noninvasive measures of atherosclerosis for incident myocardial infarction: the Rotterdam Study.. Circulation.

[8] Wilson PW, Kauppila LI, O'Donnell CJ, Kiel DP, Hannan M (2001). Abdominal aortic calcific deposits are an important predictor of vascular morbidity and mortality.. Circulation.

[9] U.S. Preventive Services Task Force (2002). Screening for osteoporosis in postmenopausal women: recommendations and rationale.. Annals of Internal Medicine.

[10] ACOG Committee on Practice (2004). ACOG practice bulletin. Clinical management guidelines for obstetrician-gynecologists. Osteoporosis. Number 50, January 2003.. Obstet Gynecol.

[11] Hodgson SF, Watts NB, Bilezikian JP, Clarke BL, Gray TK (2003). American Association of Clinical Endocrinologists medical guidelines for clinical practice for the prevention and treatment of postmenopausal osteoporosis: 2001 edition, with selected updates for 2003.. Endocr Pract.

[12] Brown JP, Josse RG (2002). 2002 clinical practice guidelines for the diagnosis and management of osteoporosis in Canada.. Cmaj.

[13] Vokes T, Bachman D, Baim S, Binkley N, Broy S (2006). Vertebral fracture assessment: the 2005 ISCD Official Positions.. J Clin Densitom.

[14] Schousboe JT, Ensrud KE, Nyman JA, Kane RL, Melton LJ (2006). Cost-effectiveness of vertebral fracture assessment to detect prevalent vertebral deformity and select postmenopausal women with a femoral neck T-score>−2.5 for alendronate therapy: a modeling study.. J Clin Densitom.

[15] Thom T, Haase N, Rosamond W, Howard VJ, Rumsfeld J (2006). Heart disease and stroke statistics–2006 update: a report from the American Heart Association Statistics Committee and Stroke Statistics Subcommittee.. Circulation.

[16] Schousboe JT, Wilson KE, Kiel DP (2006). Detection of abdominal aortic calcification with lateral spine imaging using DXA.. J Clin Densitom.

[17] Vogt TM, Ross PD, Palermo L, Musliner T, Genant HK (2000). Vertebral fracture prevalence among women screened for the Fracture Intervention Trial and a simple clinical tool to screen for undiagnosed vertebral fractures. Fracture Intervention Trial Research Group.. Mayo Clin Proc.

[18] National Insitutes of Health. NIH Image. Available at http://rsb.info.nih.gov/nih-image/. Accessed on September 1, 2006

[19] Kauppila LI, Polak JF, Cupples LA, Hannan MT, Kiel DP (1997). New indices to classify location, severity and progression of calcific lesions in the abdominal aorta: a 25-year follow-up study.. Atherosclerosis.

[20] Neal DJ (2000). Bootstrap inferences about measures of correlation.. Stata Technical Bulletin Reprints.

[21] Swets JA (1988). Measuring the accuracy of diagnostic systems.. Science.

[22] Levitzky Y, Cupples L, Murabito J, Wilson P, Kiel D (2006). Abdominal aortic calcium as a risk factor for development of intermittent claudication: the Framingham Heart Study.. Circulation.

[23] Blake GM, Naeem M, Boutros M (2006). Comparison of effective dose to children and adults from dual X-ray absorptiometry examinations.. Bone.

[24] Greenland P, Abrams J, Aurigemma GP, Bond MG, Clark LT (2000). Prevention Conference V: Beyond secondary prevention: identifying the high-risk patient for primary prevention: noninvasive tests of atherosclerotic burden: Writing Group III.. Circulation.

[25] Kuller LH, Sutton-Tyrrell K (1999). Aging and cardiovascular disease. Use of subclinical measurements.. Cardiol Clin.

[26] Greenland P, Gaziano JM (2003). Clinical practice. Selecting asymptomatic patients for coronary computed tomography or electrocardiographic exercise testing.. N Engl J Med.

[27] Oei HH, Vliegenthart R, Hak AE, Iglesias del Sol A, Hofman A (2002). The association between coronary calcification assessed by electron beam computed tomography and measures of extracoronary atherosclerosis: the Rotterdam Coronary Calcification Study.. J Am Coll Cardiol.

[28] O'Malley PG, Taylor AJ (2003). Prognostic value of coronary artery calcification.. Circulation.

[29] Jayalath RW, Mangan SH, Golledge J (2005). Aortic calcification.. Eur J Vasc Endovasc Surg.

[30] Reaven PD, Sacks J (2005). Coronary artery and abdominal aortic calcification are associated with cardiovascular disease in type 2 diabetes.. Diabetologia.

[31] Tanko LB, Christiansen C, Cox DA, Geiger MJ, McNabb MA (2005). Relationship between osteoporosis and cardiovascular disease in postmenopausal women.. J Bone Miner Res.

